# miR-125a-5p post-transcriptionally suppresses GALNT7 to inhibit proliferation and invasion in cervical cancer cells via the EGFR/PI3K/AKT pathway

**DOI:** 10.1186/s12935-020-01209-8

**Published:** 2020-04-10

**Authors:** Qinxue Cao, Ning Wang, Lu Ren, Jun Tian, Shaoqin Yang, Hailing Cheng

**Affiliations:** Department Gynecology, Huaihe Hospital of Henan University, No.8 Baobei Road, Kaifeng, 475000 Henan Province China

**Keywords:** Cervical cancer, MiR-125a-5p, GALNT7, The EGFR/PI3K/AKT pathway

## Abstract

**Background:**

The carcinogenesis and progression of cervical cancer is a complex process in which numerous microRNAs are involved. The purpose of this study is to investigate the role of miR-125a-5p in progression of cervical cancer.

**Methods:**

RT-qPCR was used to detect the expression of miR-125a-5p and GALNT7 in cervical cancer tissues and cell lines. Then, the miR-125a-5p mimic, miR-125a-5p inhibitor, GALNT7 siRNA, or/and pcDNA-GALNT7 were respectively transfected into HeLa and Caski cervical cancer cells, and Cell Counting kit-8 assay, Transwell assay and flow cytometry analysis were respectively used to observe cell proliferation, invasion and apoptosis. Subsequently, luciferase reporter gene assay was employed in confirming the target relationship between miR-125a-5p and GALNT7. MiR-125a-5p mimic or/and pcDNA-GALNT7 were transfected into the cervical cancer cells at the absence of epidermal growth factor (EGF) or not, and the pcDNA-GALNT7 was transfected into the cervical cancer cells at the absence of inhibitors of multiple kinases or not. Furthermore, the effect of miR-125a-5p on tumor growth was also studied using a xenograft model of nude mice.

**Results:**

MiR-125a-5p was down-regulated in both cervical cancer tissues and cell lines and it inhibited cell proliferation and invasion of cervical cancer cells. MiR-125a-5p directly targeted and post-transcriptionally downregulated GALNT7 that was strongly upregulated in cervical cancer tissues and cell lines. Similar to the effect of miR-125a-5p mimic, silencing GALNT7 inhibited proliferation and invasion of cervical cancer cells. In addition, miR-125a-5p overexpression could counteract both GALNT7- and EGF-induced cell proliferation and invasion. GALNT7 promoted cell proliferation and invasion by activating the EGFR/PI3K/AKT kinase pathway, which could be abated by the inhibitors of the kinases. Moreover, the role of miR-125a-5p inhibited tumor formation in cervical cancer by suppressing the expression of GALNT7 in vivo.

**Conclusion:**

In conclusion, miR-125a-5p suppressed cervical cancer progression by post-transcriptionally downregulating GALNT7 and inactivating the EGFR/PI3K/AKT pathway.

## Background

Cervical cancer is one of the most common gynecological malignant diseases among woman in the worldwide, and the majority of new cases and deaths occur in developing countries every year [1, 2]. With the development of advanced diagnosis, the morbidity of cervical cancer has decreased [3–5]. However, the occurrence and development of cervical cancer is as complex as a network system, and the underlying mechanisms remain largely unknown, so the prognosis of cervical cancer also is poor [2, 6, 7]. Therefore, it is important to explore the effective therapeutic strategies.

MiRNAs are non-coding, endogenous and conserved RNAs containing 19–25 nucleotides in length [8, 9]. Numerous studies have reported that miRNAs could post-transcriptionally downregulate the expression of their matched target genes via interaction with the 3′-untranslated regions (3′-UTRs) of mRNA, causing mRNA degradation or interference translation [10, 11]. Therefore, miRNAs are involved in various cellular biological processes, including cell growth, invasion, development, and apoptosis [12–14]. Several research reported that miRNA-125a-5p level was decreased in many tumor tissues, compared to the adjacent normal tissues [15–17], and some studies had proved that miR-125a-5p could repress cell proliferation and invasion, suggesting that miR-125a-5p might act as a tumor inhibitor [18–21]. However, the underlying mechanism in cervical cancer of miR-125a-5p is still not particularly clear.

As one member of the UDP-*N*-acetyl-α-d-galactosamine:polypeptide N-acetylgalactosaminyltransferase (GalNAc-T or GALNT) family, GALNT7 acts as a glycosyltransferase in protein O-GlcNAcylatio, regulating the interaction between cancer cells and the extracellular environment [22–24]. Previous studies had demonstrated that aberrant glycosylation could promote cell growth, transformation, metastasis, apoptosis, migration and differentiation [25–27]. GALNT7 expression is on the rise in multiple types of malignant tumors, suggesting that GALNT7 is involved in the occurrence and development of tumors [28, 29]. The research also reported that inhibiting GALNT7 expression might contribute to tumor regression following steroid androgen hormones depletion therapy [30]. Li Yang et al. reported that LncSNHG7 increased the level of GALNT7 to promote the progression of colorectal cancer [31]. Several studies have shown that miRNAs also could regulate the expression of GALNT7 [32, 33]. However, the interaction between miR-125a-5p and GALNT7 in cervical cancer is unclear.

In this study, the results indicated that the expression of miR-125a-5p was significantly lower than that in cervical cancer tissues and cell lines. And miR-125a-5p played a cancer suppressor gene role by directly bounding to GALNT7 to repress the expression of GALNT7 and participated in the regulation of cervical cancer progression. GALNT7 promoted cell proliferation and invasion by activating the EGFR/PI3K/AKT pathway. Therefore, we speculated that miR-125a-5p contributed to cervical cancer development and progression and could be a potential biomarker for the diagnosis and treatment of cervical cancer.

## Materials and methods

### Clinical specimens

Cervical cancer tissues samples and their corresponding adjacent tissues were obtained from twenty patients (mean age, 51.75 ± 10.43 years; age range, 33–72 years) with cervical cancer in the Huaihe Hospital of Henan University (Kaifeng, China) after surgical resection from June 2017 to May 2018. All the histological diagnoses for cervical cancer and adjacent tissues were reviewed and recognized by 2 pathologists independently. None of patients was treated with chemoradiotherapy prior the surgery. The research had got the informed consent by each patient and certified by the Ethics Committee.

### Antibodies and inhibitors

GALNT7 (ab97645), EGFR (ab32077), PI3K (42555), p-PI3K (17366), AKT (9272S), p-AKT (9611S), goat antirabbit IgG-HRP and goat anti-mouse IgG-HRP antibody were purchased from Abcam (Cambridge, UK). GAPDH was obtained from Santa Cruz Biotechnology (Santa Cruz, CA). PD153035 (CalbioChem, San Diego, CA) and LY294002 (Cell Signaling Technology, USA) were pre-incubated with cells for 1 h at the indicated concentrations.

### Cell culture and transfection

The human cervical cancer cell lines HeLa and Caski were purchased from American Type Culture Collection (ATCC, Manassas, VA, USA). The cells were cultured in RPMI 1640 medium (Gibco, Rockville, MD) containing 10% fetal bovine serum and 100 U/ml penicillin and 100 μg/mL streptomycin (Sigma, St. Louis, MO, USA) with 5% CO_2_ at 37 °C. MiR-125a-5p mimic, negative control oligonucleotides (NC-mimic), GALNT7 pcDNA3.1 vector (pcDNA-GALNT7) and empty vector (Vector) were purchased from RiboBio Co., Ltd (Guangzhou, China) and transfected into cells using RiboBio Transfection Kit (RiboBio Co., Ltd). Small interfering RNA of GALNT7 (si-GALNT7) and scramble siRNA of GALNT7 (Scramble) were purchased from Santa Cruz Biotechnology (Santa Cruz, CA, USA). All oligos and plasmids were transfected into cells by using Lipofectamine 3000 Transfection Reagent (Invitrogen, Carlsbad, CA, USA) according to the manufacturer’s instructions.

### Reverse transcription-quantitative polymerase chain reaction (RT-qPCR)

Total RNA was isolated from tissues and cells using Trizol reagent (Invitrogen, Carlsbad, CA, USA). For the detection of miRNA, real time quantitative polymerase chain reaction was performed using the TaqMan microRNA Reverse Transcription Kit (Applied Biosystems, Foster City, CA, USA) and following parameter values to program the thermal cycler: 16 °C for 30 min, 42 °C for 30 min and 85 °C for 5 min. U6 was served as the internal control for the normalization of miRNA expression. For the detection of mRNA, reverse transcription was performed using M-MLV Reverse Transcriptase (Takara Biotechnology, Dalian, China) and cDNA amplification was carried out using the SYBR Green PCR Master Mix (Applied Biosystems). PCR amplification was carried out using an Applied Biosystems 7900HT Fast Real-Time PCR System following the thermal cycling conditions: 95 °C for 10 min; 40 cycles of 95 °C for 15 s and 60 °C for 60 s. GAPDH was used as the internal control for normalizing mRNA expression and 2^−(ΔΔCt)^ method was applied to calculate the fold change. Primers were as follows: GALNT7 forward: 5′-TGCTGGAGGAGATTCCCA GAA-3′, GALNT7 reverse: 5′-GCACAGGATCATGGTAGGTG AA-3′; miR-125a-5p forward: 5′-TGAGACCCTTTAACCTGTGA-3′, miR-125a-5p reverse: 5′-GCGAGCACAGAATTAATACGAC-3′; U6 forward: 5′-CTCGCTTCGGCAGCACA-3′, U6 reverse: 5′-AACGCTTCACGAATTTGCGT-3′; GAPDH forward: 5′-TGTGGGCATCAATGG ATTTGG-3′, GAPDH reverse: 5′-ACACCATGTATTCCGGGTCAAT-3′.

### Analysis of cell proliferation

The Cell Counting Kit-8 (CCK-8; Beyotime Biotechnology, Shanghai, China) was used to assess cell proliferation. In brief, cells were seeded into 96-well plates and cultured for 24 h. After transfection for 24 h, 48 h or 72 h, 10 μL of CCK-8 solution was added and incubated for another 2 h at 37 °C. The absorption values at 450 nm were detected with a micro-plate analyzer (Molecular Devices, Sunnyvale, CA, USA).

### Invasion assays

24-well Transwell chambers were used to perform invasion assays. Cells were seeded into the Matrigel-coated upper chamber. The medium containing 10% FBS was added to the lower chamber. After 24 h of incubation, the no migration cells were carefully removed and the membranes were fixed with 4% paraformaldehyde and stained with 0.1% crystal violet. The invasion average cells number was calculated under a microscope.

### Cell apoptosis

Cell apoptosis was determined by using flow cytometry. After harvested, cells were washed with phosphate-buffered saline for three times. Cells were resuspended in the buffer with 5 μL Annexin V-FITC and 5 μL propidium iodide at room temperature in the dark for 20 min, cell apoptosis rate were examined by using flow cytometry (BD Biosciences, USA).

### Luciferase reporter gene assay

The putative relationship between miR-125a-5p and GALNT7 were predicted using TargetScan (http://www.targetscan.org/). The wild and mutant types of GALNT7 mRNA 3′-UTR fragment including the predicted miR-125a-5p binding sites was amplified and constructed into the pcDNA3.1 (+) vector (Invitrogen, Carlsbad, CA, USA). Cells were seeded into 24-well plates, and co-transfected with the constructs and miR-125a-5p mimic for 48 h. Luciferase reporter activity was measured using Dual-Luciferase Reporter Assay System (Promega, Madison, WI) under the manufacturer’s instructions.

### Western blot analysis

Cells were harvested and lysed in the RIPA buffer for 30 min at 4 °C. The protein concentrations were determined using BCA Protein Assay Kit (Beyotime Biotechnology, Shanghai, China). Proteins were loaded onto 10% SDS–polyacrylamide gel to separate the proteins. Then, the separated proteins were transferred to a PVDF membrane and the membrane was blocked with mixed liquor containing 5% skim milk in TBST and 0.1% Tween 20 for 1 h at room temperature. Afterward, the membrane was incubated with primary antibodies against GALNT7 (1:500 dilution), EGFR (1:500 dilution), PI3K (1:1000 dilution), p-PI3K(1:1000 dilution), AKT(1:1000 dilution), p-AKT(1:1000 dilution) and GAPDH (1:2000 dilution). HRP-conjugated secondary antibodies (1:2000 dilution) were incubated for 1 h after washing with TBST. Proteins were detected using enhanced chemiluminescence reagents (Thermo Fisher Scientific, Inc.). Set the exposure time of proteins to about 40 s. The protein bands images were quantified with Image-Pro Plus 6.0 software.

### Nude mice xenograft model

Tumor formation was performed by establishing a xenograft model. The experiments involving animals were approved by the Ethics Committee for the Use and Care of Animals of Henan University (Kaifeng, China). A total of 15 BALB/c female nude mice (age:4 weeks old; weight: 12.7 ± 0.4 g;) were purchased from Institute of Laboratory Animals in Chinses Academy of Medical Sciences (Shanghai, China) and randomly divided into the control group, NC-mimic group and miR-125a-5p mimic group. HeLa cells stably expressing miR-125a-5p mimic or NC-mimic were injected subcutaneously into the left axilla of each mice at a concentration of 5 × 10^7^ cells/mL in cold PBS (100 μL every mouse). Thirty days later, the mice were sacrificed and the cancer tissues were harvested. Excised tumors were evaluated for volume and weight. The tumor volume was calculated every 5 days by the formula (mm^3^): tumor volume = length × width^2^ × 0.5. The level of miR-125a-5p in tumors was detected using RT-qPCR and the protein level of GALNT7 was analyzed using western blot.

### Statistical analysis

All data represent as the mean ± standard error of mean (SEM). Student’s t test or one‐way analysis of variance (ANOVA) was analyzed by differences between experimental groups. *P < 0.05 and **P < 0.01 were considered to be statistically significant.

## Results

### MiR-125a-5p is downregulated in cervical cancer tissues and cell lines

**Fig. 1:**
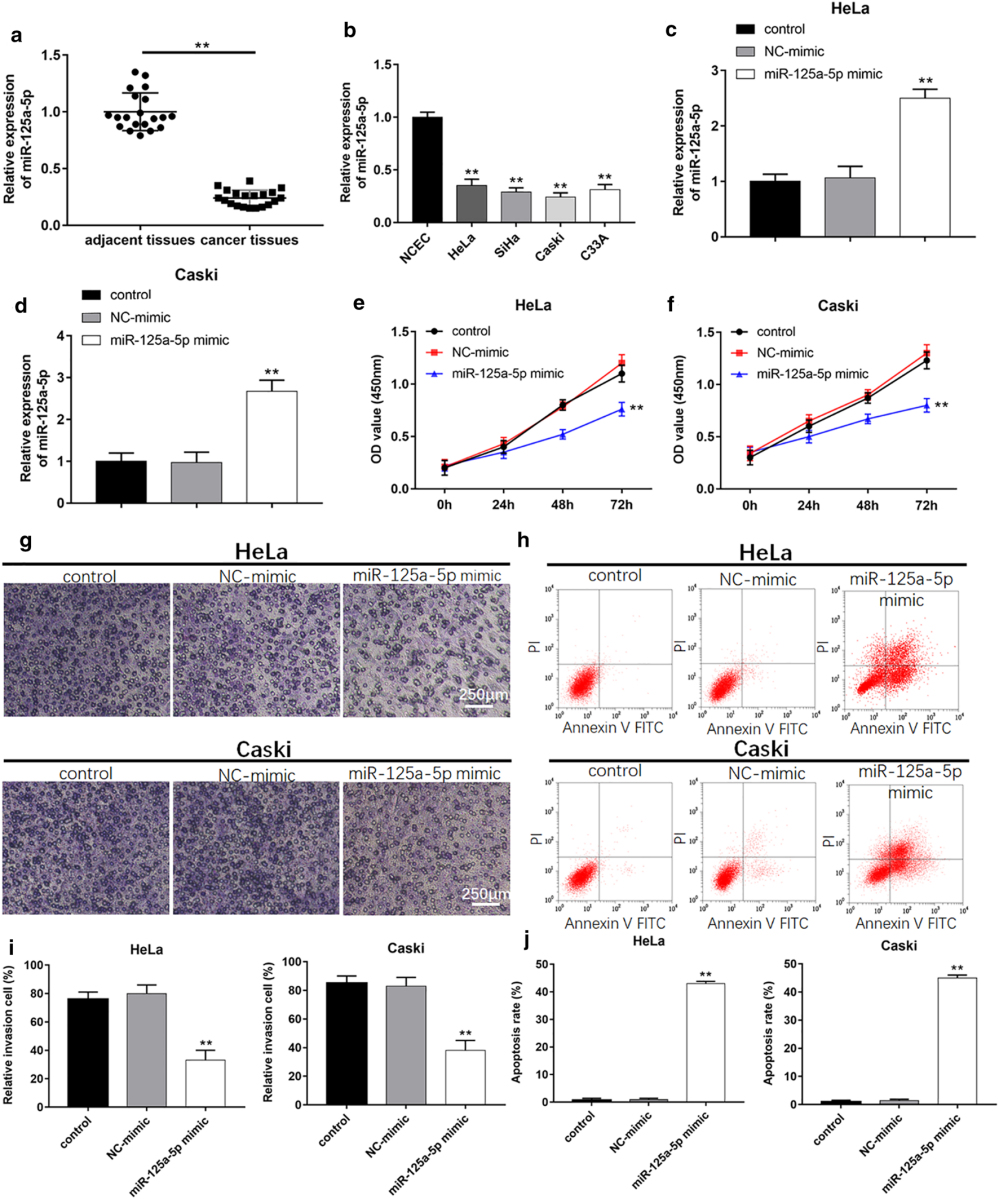
Overexpression of miR-125a-5p suppresses cell proliferation, invasion and increases cell apoptosis. **a** Relative expression of miR-125a-5p in cervical cancer tissues in comparison with normal cancer adjacent tissues was analyzed (n = 20). **b** MiR-125a-5p levels in cervical cancer lines including HeLa, SiHa, Caski and C33A were significantly decreased compared with that in the normal cervical epithelial cells (NCEC). **c**, **d** Transfection efficiency of HeLa and Caski cells was detected using RT-qPCR after transfection with miR-125a-5p mimic. **e**, **f** Cell proliferation was analyzed using CCK-8 assay after transfecting with NC-mimic or miR-125a-5p mimic for 24 h, 48 h and 72 h. **g**, **i** Transwell was used to evaluate cell migration after cells were transfected with miR-125a-5p mimic for 24 h. **h**, **j**Apoptosis rates of cervical cancer after transfection with miR-125a-5p mimic were detected using Flow cytometry. * *P *< 0.05, ** *P *< 0.01

In order to explore the role of miR-125a-5p, we first examined the expression of miR-125a-5p in cervical cancer tissues and adjacent tissues using RT-qPCR. The results revealed that the level of miR-125a-5p was significantly lower than that in adjacent tissues (Fig. 1a, *P *< 0.01). Moreover, we tested the miR-125a-5p expression in the normal cervical epithelial cells (NCEC) and four cervical cancer cells, including HeLa, SiHa, Caski and C33A. Our results showed that the levels of miR-125a-5p were downregulated in cervical cancer cells compared with normal epithelial cells (Fig. 1b, *P *< 0.01). The data indicated that miR-125a-5p could be involved in cervical carcinogenesis.

### MiR-125a-5p suppresses cell proliferation, invasion and increases cell apoptosis in vitro

To verity the function of miR-125a-5p, we transfected miR-125a-5p mimic and NC-mimic into HeLa and Caski cells and detected the expression levels of miR-125a-5p. The results showed that miR-125a-5p levels were markedly upregulated after transfection with miR-125a-5p mimic in comparison with NC-mimic group (Fig. 1c, d, *P *< 0.01). The proliferative capability was detected using CCK-8 assay. Overexpression of miR-125a-5p significantly inhibited the proliferation of HeLa and Caski cells (Fig. 1e, f, *P *< 0.01). Moreover, the Transwell assay showed that overexpression of miR-125a-5p could reduce the number of cell invasions compared with control and NC-mimic groups (Fig. 1g, i, *P *< 0.01). In addition, the apoptotic cells were also significantly more than those in control and NC-mimic groups after upregulation of miR-125a-5p. (Figure 1h, j, *P *< 0.01). These results demonstrated that miR-125a-5p regulated cell proliferation, invasion and apoptosis in cervical cancer.

### GALNT7 is a target gene of miR-125a-5p in cervical cancer cells

**Fig. 2:**
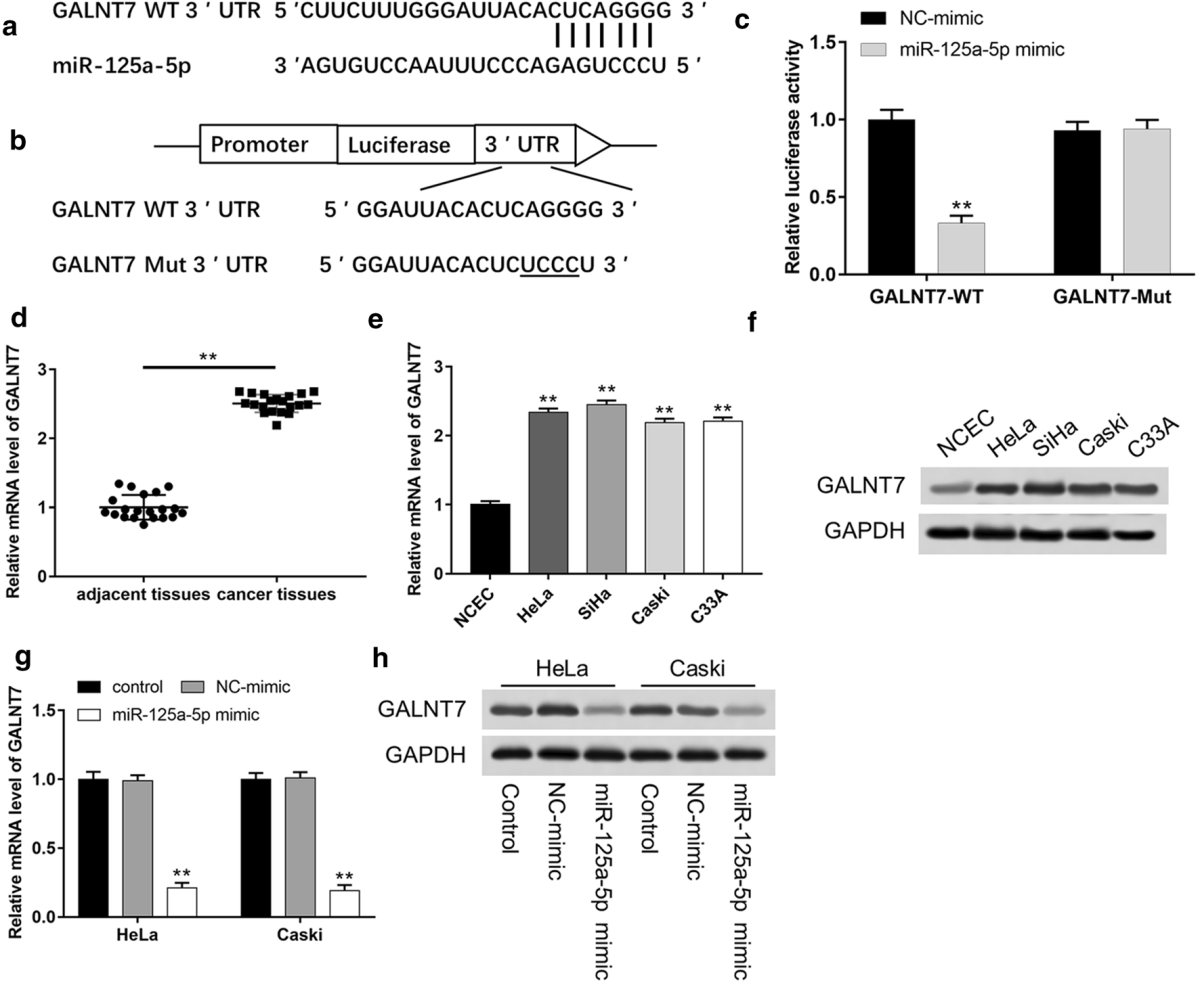
GALNT7 is a target gene of miR-125a-5p in cervical cancer cell lines. **a** Bioinformatic analysis predicts the targeting site of miR-125a-5p in GALNT7 mRNA 3′-UTR. **b** The sequence alignments of GALNT7 in the wild-type and mutant-type were shown. **c** The luciferase reporter plasmid containing the WT or MUT GALNT7 3′-UTR was co-transfected into HEK293T cells with miR-125a-5p mimic or NC-mimic. The relative luciferase activity was tested at 48 h after co-transfection. **d** GALNT7 expression in adjacent normal and cervical cancer tissues were determined using RT-qPCR (n = 20). The mRNA **e** and protein **f** levels of GALNT7 in HeLa, SiHa, Caski and C33A cervical cell lines and NCEC were detected using RT-qPCR and western blot, respectively. The mRNA **g** and protein **h** levels of GALNT7 were detected after transfection with NC-mimic or miR-125a-5p mimic. *P < 0.05, **P < 0.01

MiR-125a-5p had a prominent effect on inhibiting cervical cancer cells, however, the potential mechanism was still unclear. Bioinformatics software was used to analyze the underlying target gene of miR-125a-5p. The result showed that the 3′-UTR of GALNT7 mRNA existed a targeting site for miR-125a-5p (Fig. 2a). To further investigate whether miR-125a-5p could directly target the 3′-UTR of GALNT7, the luciferase reporter gene assay containing wild-type (WT) or mutant-type (Mut) binding site showed that overexpression of miR-125a-5p significantly reduced the luciferase activity of wild-type GALNT7 3′-UTR, but failed to effect the mutant-type (Fig. 2b, c *P *< 0.01). Moreover, we also detected the levels of GALNT7 in cervical cancer tissues and cervical cancer cell lines. As shown in Fig. 2d, the mRNA level of GALNT7 significantly increased in cervical cancer tissues compared with adjacent tissues (*P *< 0.01). And the mRNA and protein levels of GALNT7 in four cervical cancer cell lines were also higher than those in NCEC cells (Fig. 2e, f). When HeLa and Caski cells were transfected with miR-125a-5p mimic, the mRNA and protein levels of GALNT7 markedly reduced in contrast with the control and NC-mimic groups (Fig. 2g, h). Overall, these results suggested that GALNT7 was a target gene of miR-125a-5p and miR-125a-5p could regulate GALNT7 expression.

### Silence of GALNT7 inhibits proliferation and invasion ability of cervical cancer cells

**Fig. 3:**
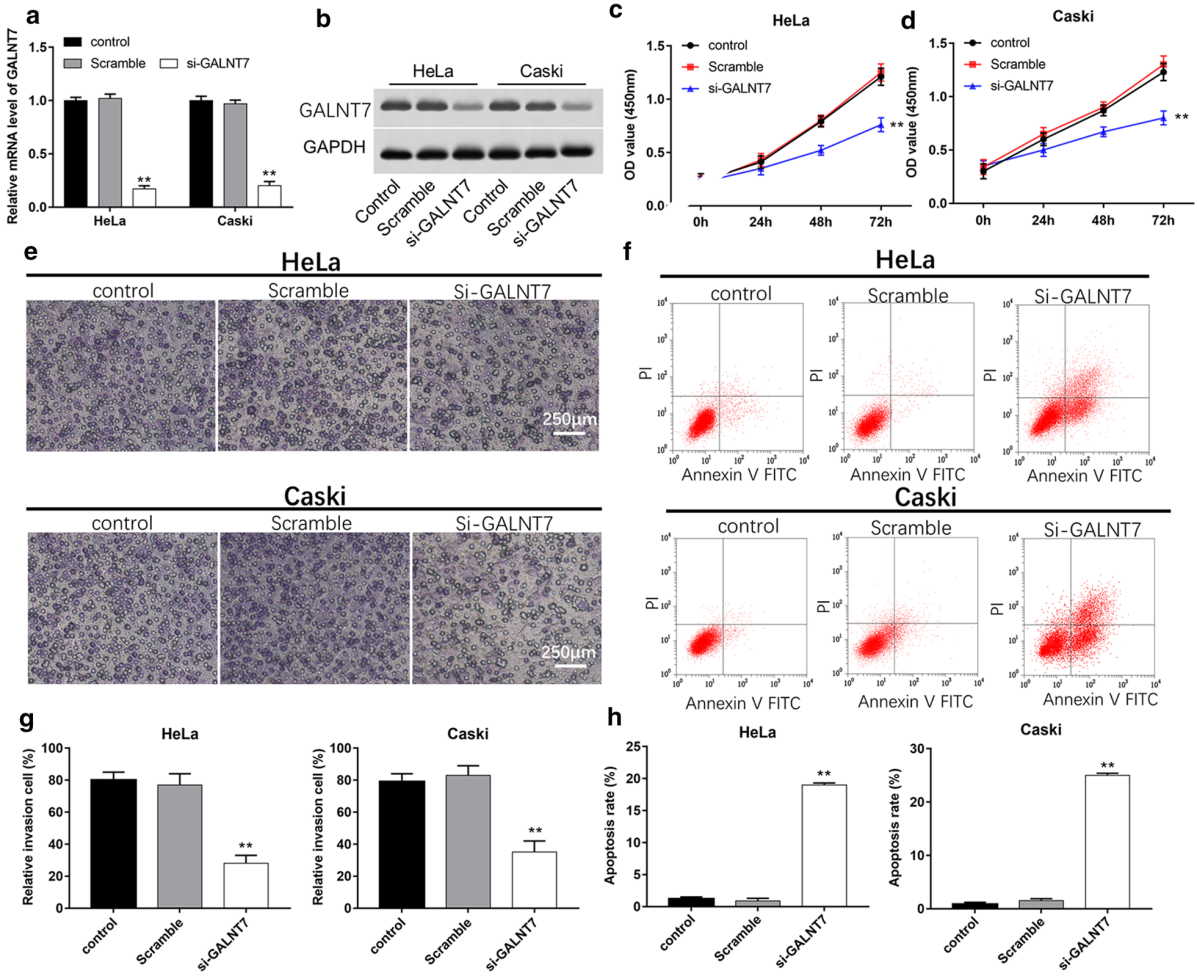
Silence of GALNT7 mediates cell proliferation, invasion and apoptosis of HeLa and Caski cell in vitro. The mRNA **a** and protein **b** levels of GALNT7 were detected after transfection with Scramble or si-GALNT7. **c**, **d** Cell proliferation was analyzed using CCK-8 assay after transfection with si-GALNT7 for 24 h, 48 h and 72 h. **e**, **g** Cells were transfected with si-GALNT7 for 24 h, and the effect of silencing GALNT7 on HeLa and Caski cell invasion was evaluated using Transwell invasion assay. **f**, **h** Apoptosis rates of HeLa and Caski cells after transfecting with si-GALNT7 for 24 h were detected using Flow cytometry. *P < 0.05, **P < 0.01

Aberrant glycosylation is a distinguishing feature of cancer, and is related to cell proliferation, adhesion, transformation and metastasis. Overexpression of GALNT7 can facilitate this process. Therefore, we used the small interfering RNA to silence the expression of GALNT7 and observed the effect on cells. As shown in Fig. 3a, b, the mRNA and protein levels of GALNT7 remarkably decreased on cells after transfection with si-GALNT7. Compared with Scramble, the proliferation ability was significantly inhibited in HeLa and Caski cells. (Figure 3c, d, *P *< 0.01). To measure invasion ability of cervical cancer, the Transwell assays were utilized. The relative number of cell invasion decreased in HeLa and Caski cells after transfection with si-GALNT7 relative to control and Scramble groups (Fig. 3e, g, P < 0.01). Moreover, downregulation of GALNT7 could result in more apoptotic cells than those in control and Scramble groups (Fig. 3f, h, P < 0.01). Thus, we concluded that knockdown of GALNT7 played tumor-suppressive role on cell proliferation and invasion.

### MiR-125a-5p overexpression counteracts the cancer promotion effect of GALNT7 in cervical cancer cells

**Fig. 4:**
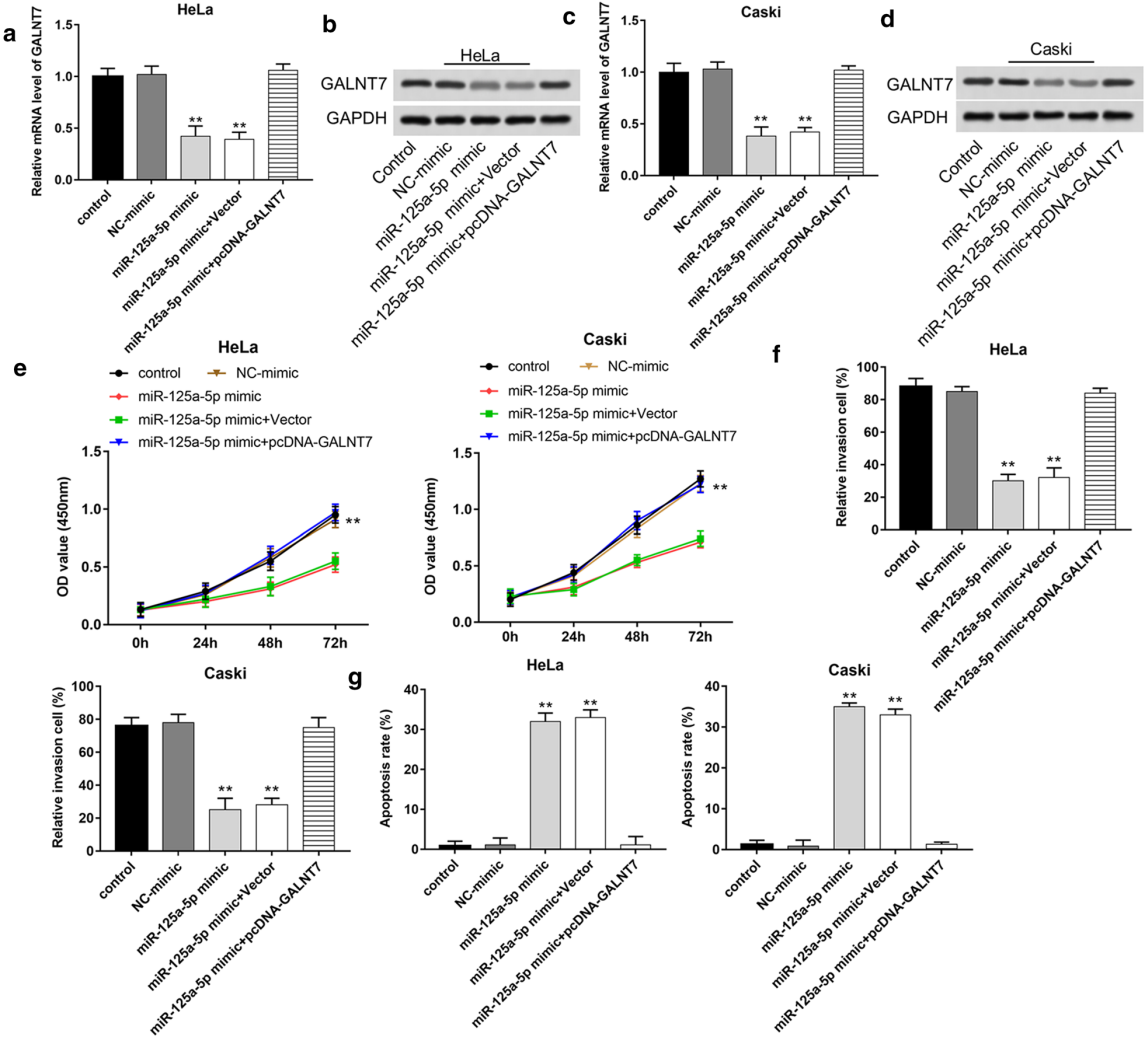
MiR-125a-5p overexpression counteracts the cancer promoting effect induced by GALNT7 in cervical cancer cell lines. HeLa and Caski cells were transfected with NC-mimic, miR-125a-5p mimic, or co-transfected with miR-125a-5p mimic and Vector, miR-125a-5p mimic and pcDNA-GALNT7. The mRNA **a**, **c** and protein **b**, **d** levels of GALNT7 in HeLa and Caski cells were detected using RT-qPCR and western blot, respectively. **e** Cell proliferation was measured using the CCK-8 assay after transfection for 24 h. **f** Cell invasion was detected using the Transwell invasion assay after transfection for 24 h. **g** Cell apoptosis was measured using Flow cytometry after transfection for 24 h. *P < 0.05, **P < 0.01

We performed the rescue experiments on the cervical cancer cell proliferation, invasion and apoptosis to confirm the effects of miR-125a-5p. As shown in Fig. 4a–d, the mRNA and protein levels of GALNT7 decreased when HeLa and Caski cells were transfected with miR-125a-5p mimic, while increased after co-transfected with pcDNA-GALNT7 and miR-125a-5p mimic (*P *< 0.01). After 96 h incubation, the absorbance of cells transfected with miR-125a-5p mimic was lower than that in control and co-transfected with pcDNA-GALNT7 and miR-125a-5p mimic groups (Fig. 4e, *P *< 0.01). Moreover, invasion assay showed that transfection with the pcDNA-GALNT7 counteracted the decrease in the number of cell invasion induced by miR-125a-5p mimic (Fig. 4e, f, *P *< 0.01). Cells transfected with miR-125a-5p mimic could increase cell apoptosis rate and co-transfected with pcDNA-GALNT7 and miR-125a-5p mimic reversed the increase effects (Fig. 4g, h, *P *< 0.01). These results indicated that restoration of miR-125a-5p counteracted the effects of GALNT7 expression in HeLa and Caski cells.

### MiR-125a-5p inhibits EGF-induced cell proliferation and invasion

**Fig. 5:**
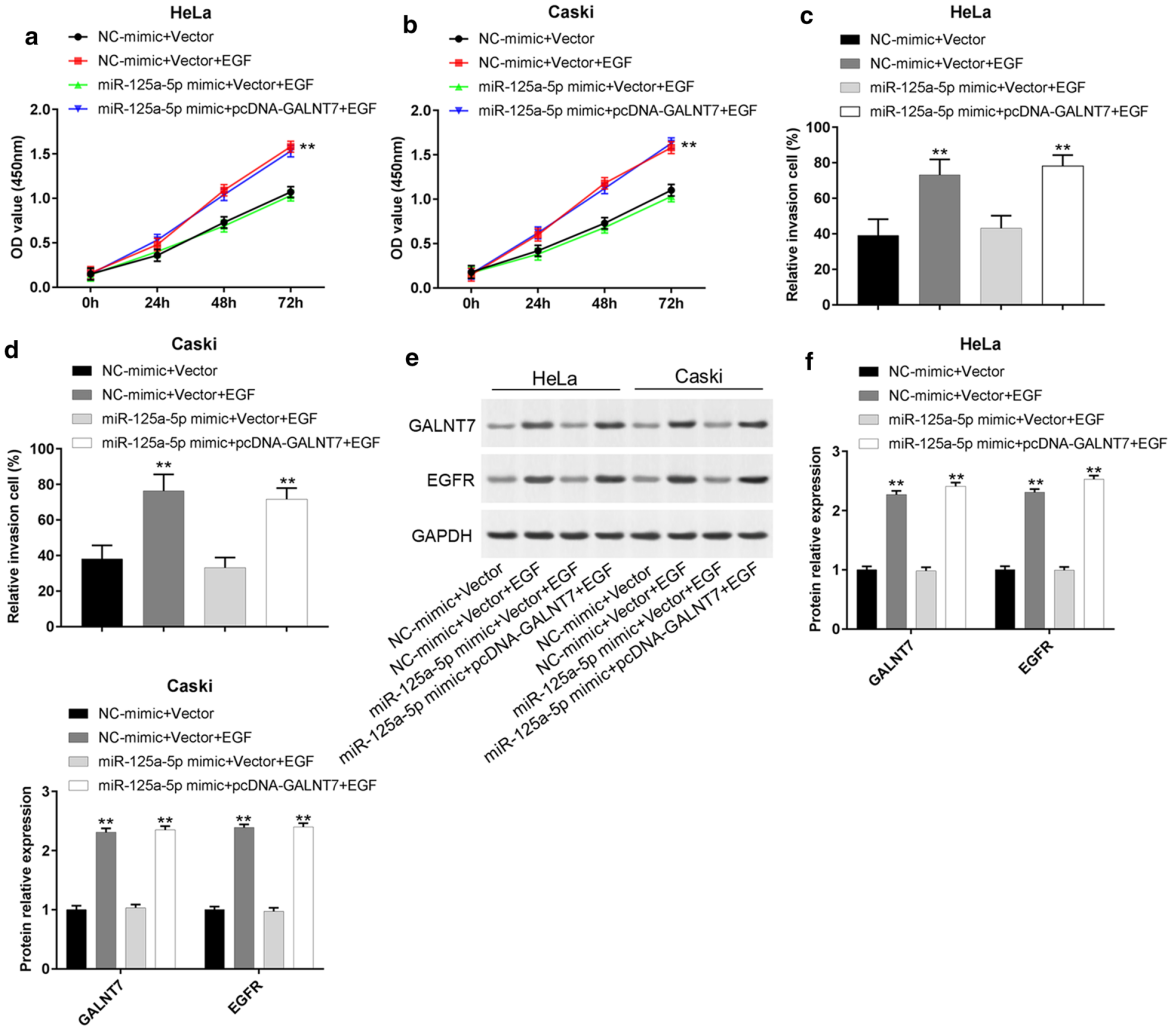
MiR-125a-5p inhibits EGF-induced cell proliferation and invasion. Cells were treated with 100 ng/mL EGF for 24 h before transfecting with NC-mimic and Vector, miR-125a-5p mimic and Vector, or miR-125a-5p mimic and pcDNA-GALNT7. **a**, **b** HeLa and Caski cell proliferation was measured using CCK-8 assay after transfection for 24 h, 48 h and 72 h. **c**, **d** Cell invasion was evaluated using Transwell invasion assay after transfection for 24 h. **e**, **f** The protein levels of GALNT7 and EGFR in HeLa and Caski cells were detected using western blot. *P < 0.05, **P < 0.01

To further investigate the effect of miR-125a-5p on HeLa and Caski cells, EGF-induced cell assay was detected. Epidermal growth factor (EGF), a noted growth factor, induces changes in the molecules of cell–cell junction and eventually leads to cell proliferation and invasion [34–36]. As shown in Fig. 5a–d, the cell proliferation and invasion capacity of NC-mimic and Vector and EGF group significantly increased after treating with 100 ng/mL EGF for 24 h compared with NC-mimic and Vector group (P < 0.01). However, overexpression of miR-125a-5p suppressed EGF-induced cell proliferation and invasion. Upregulation of GALNT7 could counteract the inhibitory effect of miR-125a-5p on cell proliferation and invasion (P < 0.01). Western blot assay showed that EGF induction can increase the protein expression of GALNT7 and EGFR compared with without EGF treatment, while miR-125a-5p could inhibit the increasing effect of GALNT7 and EGFR (Fig. 5e, f, P < 0.01). Overexpression of GALNT7 could reverse those inhibitory effect. These findings revealed that miR-125a-5p could inhibit EGF-induced cell proliferation and invasion by inhibiting the protein expression of GALNT7 and EGFR.

### GALNT7 promotes cell proliferation and invasion by activating EGFR/PI3K/AKT pathway

**Fig. 6:**
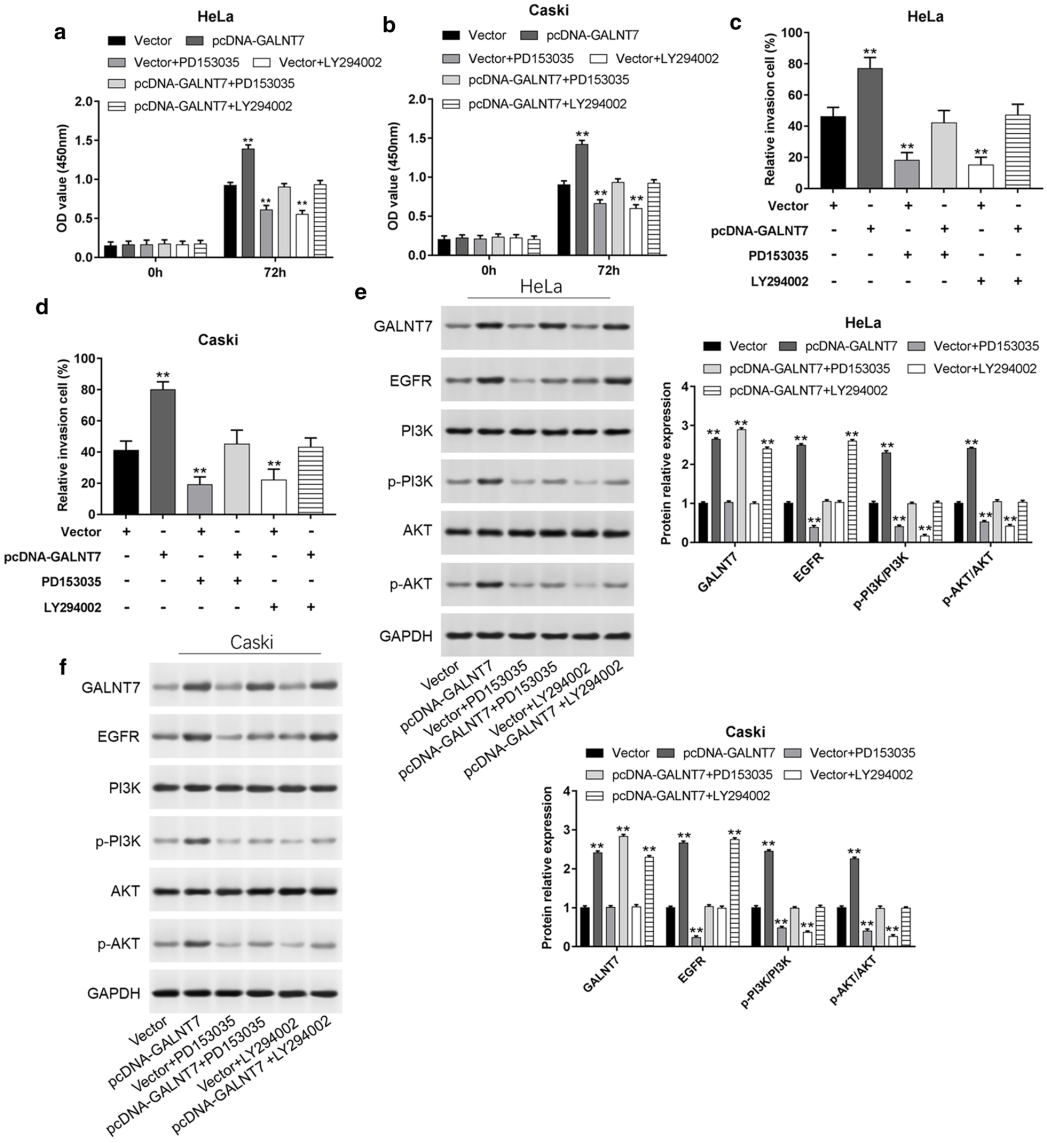
GALNT7 promotes cell proliferation and invasion by activating EGFR/PI3K/AKT pathway. HeLa and Caski cells were transfected with Vector, pcDNA-GALNT7, or treated with 10 μM PD153035 and transfected with Vector or pcDNA-GALNT7, or treated with 10 μM LY294002 and transfected with Vector or pcDNA-GALNT7 for 24 h. **a**, **b** Cell proliferation was detected using CCK-8 assay after transfection for 72 h. **c**, **d** Transwell invasion assay was used to evaluate the invasion capacity after transfection for 24 h. **e**, **f** western blot analysis was used to detect the protein expression levels of GALNT7, EGFR, PI3K, p-PI3K, AKT, p-AKT after transfection for 24 h in HeLa and Caski cells, respectively. *P < 0.05, **P < 0.01

It has been reported that EGFR/PI3K/AKT pathway was one of the most enriched pathways involved in the development of cervical cancer [37, 38], so we investigated the role of EGFR/PI3K/AKT pathway in the development of GALNT7-mediated cervical cancer. Epidermal growth factor receptor (EGFR) kinase inhibitor PD153035 (10 μM) and phosphatidylinositol 3 kinase (PI3K) inhibitor LY294002 (10 μM) were utilized in the experiments. As shown in Fig. 6a, b, compared with Vector group, cell proliferation was significantly increased when cells were transfected with pcDNA-GALNT7 (*P *< 0.01), but the proliferation ability significantly decreased after PD153035 or LY294002 treatment (*P *< 0.01). When cells were transfected with pcDNA-GALNT7 and simultaneously treated with PD153035 or LY294002, the proliferation capacity did not change significantly, indicating that blocking the pathway could inhibit cell proliferation. And we can see the similar effect in cell invasion assay (Fig. 6c, d). Cell invasion number was higher than Vector group when cells were transfected with pcDNA-GALNT7(*P *< 0.01) and after the pathway was blocked by PD153035 or LY294002, the cell invasion ability decreased again (*P *< 0.01). We also detected the expression levels of GALNT7, EGFR, PI3K, p-PI3K, AKT and p-AKT in HeLa and Caski cells after transfecting with pcDNA-GALNT7 or treating with PD153035 and LY294002. The results showed that high levels of GALNT7, EGFR, p-PI3K and p-AKT were observed when cells transfected with pcDNA-GALNT7 as shown in Fig. 6e, f. Compared with Vector group, cells treated with PD153035 could significantly inhibit the expression of EGFR, p-PI3K, p-AKT and cells treated with LY294002 could significantly inhibit the level of p-PI3K and p-AKT. After blocking the pathway with PD153035 or LY294002, overexpression of GALNT7, EGFR, p-PI3K, p-AKT did not increase. Overall, these data suggested that GALNT7 played an important role in cervical cancer progression via EGFR/PI3K/AKT pathway.

### Overexpression of miR-125a-5p suppressed tumor growth of cervical cancer and GALNT7 expression in vivo

**Fig. 7:**
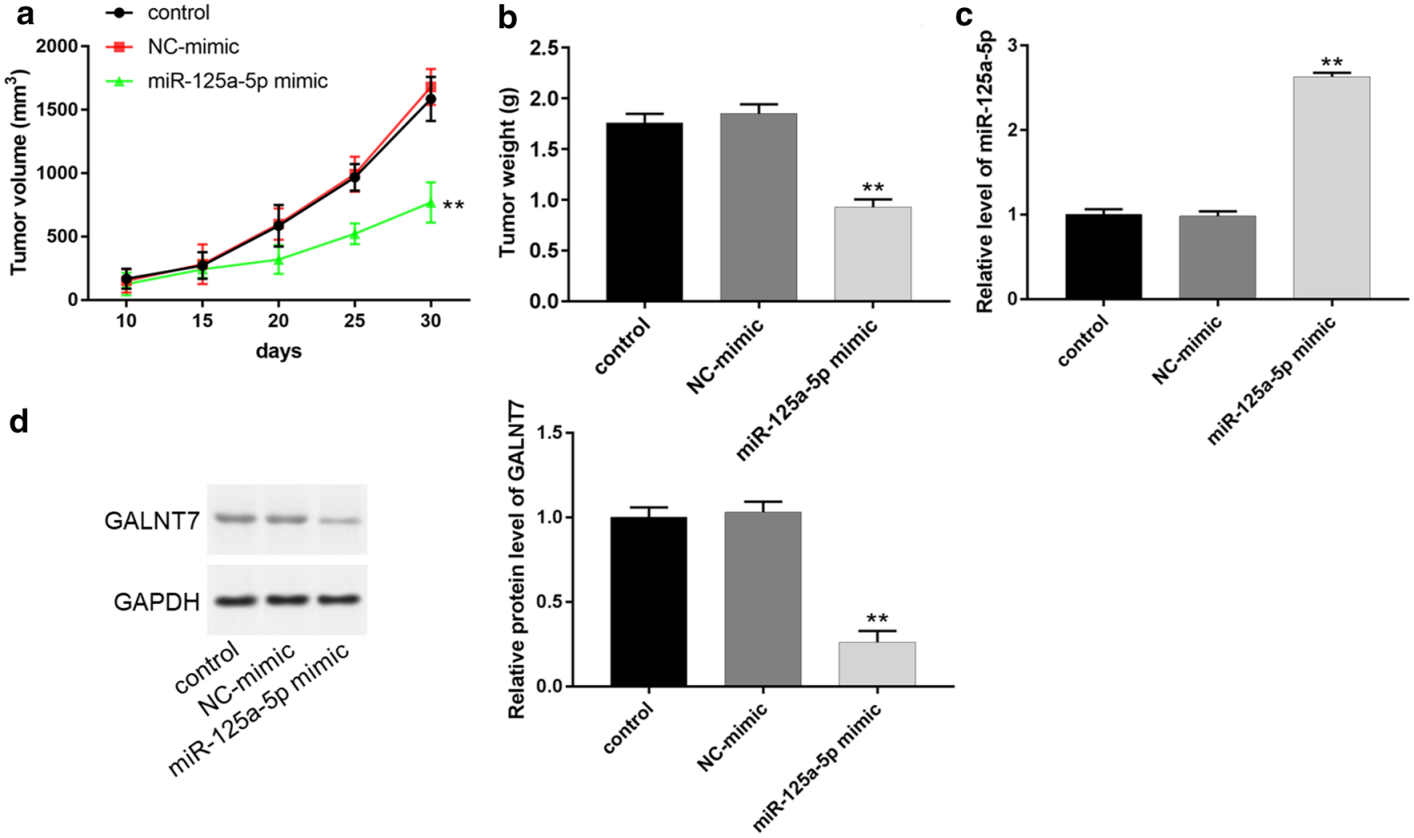
Overexpression of miR-125a-5p suppressed tumor growth of cervical cancer and GALNT7 expression in vivo. **a** Tumor volume of subcutaneous xenograft tumor model developed from miR-125a-5p overexpressed HeLa cells. **b** Tumor weight of three different groups were compared. **c** The levels of miR-125a-5p in xenografts were detected using RT-qPCR. **d** The protein expression of GALNT7 in xenografts was analyzed using western blot. **P < 0.01

In order to investigate whether miR-125a-5p had antitumor effect in vivo, we established a xenograft tumor model. The results showed that the size and weight of tumors in miR-125a-5p mimic group were significantly smaller than those of control and NC-mimic groups (Fig. 7a, b, *P *< 0.01). Moreover, in relation to the control and NC-mimic groups, the level of miR-125a-5p in tumor increased, while the protein expression of GALNT7 decreased in miR-125a-5p mimic group (Fig. 7c, d, *P *< 0.01). All results suggested that miR-125a-5p could inhibit the growth of cervical cancer in vivo by suppressing the expression of GALNT7.

## Discussion

It has been reported that abundant miRNAs are closely related to the genesis and development of cervical cancer [39, 40]. Aberrant miRNAs expression may contribute to tumor progression. Recent investigations showed that with the development of cervical cancer, the levels of miR-338-3p [5], miR-187 [6], and miR-433 [1] significantly decreased, but miR-221-3p expression upregulated [3]. Downregulation of miR-125a-5p had been revealed in colorectal cancer, gastric cancer, bladder cancer, and osteosarcoma [16, 41–43]. Our study found that miR-125a-5p levels also were downregulated in cervical cancer tissues, demonstrating that it could be connected with cervical cancer. Moreover, overexpression of miR-125a-5p impeded cell proliferation and invasion.

Accumulated studies showed that many miRNAs could act as tumor suppressors by mediating their target genes and miRNAs also were acquainted with the function to bind to mRNA 3′UTRs of target genes which induced mRNA degradation or regulated their expression post-transcriptionally. Several target genes of miR-125a-5p had been reported, for example, miR-125a-5p suppressed bladder cancer progression through targeting FUT4 [44], inhibited colorectal cancer invasion and migration by targeting TAZ [45] and so on. In our study, GALNT7 was found as a downstream target of miR-125a-5p and participated in the metabolism of cervical cancer. GALNT7 acted as a glycosyltransferase in protein O-GlcNAcylation, and it was highly expressed in many tumors. For example, overexpression of GALNT7 enhanced hepatocellular carcinoma cell proliferation and migration [46]. GALNT7 also acted as a key contributor of the pro-metastatic effects in laryngeal carcinoma cells [47]. Our results showed that GALNT7 also highly expressed in cervical cancer cells, and downregulation of GALNT7 could inhibit tumor proliferation and invasion. Moreover, overexpression of miR-125a-5p can neutralize the effect of GALNT7 on tumor proliferation and invasion.

Previous studies found that GALNTs, which were transported to the Golgi, were activated by tyrosine kinase Src and the GALNTs activation pathway could induce high Tn levels and was a key driver of tumor growth [48]. Further study revealed that growth factors such EGF also could induce this relocation, and in accordance with the mode of activation of Src [49], so we also study the effect of EGF induction of cells. In this study, cell proliferation and invasion were increased under EGF stimulation, and overexpression of miR-125a-5p significantly reversed EGF-induced proliferation and invasion of cervical cancer cell lines.

However, the underlying mechanism by which GALNT7 promoted tumor progression need further study. Recent study revealed that inhibiting the expression of GALNT7 in melanoma cells could decrease synthesis of immunosuppressive cytokine IL-10 and enhance immune cell activation and recruitment [50]. In glioma cells, GALNT7 was co-expressed with several gene including STAT3 which was associated with the JAK-STAT signaling pathway, PVR which participated in the CAMs pathway. These suggested that GALNT7 promoted cancer development by activating the JAK-ATAT or CAMs signaling pathway [27, 51].Earlier studies also showed that EGF was involved in cell proliferation through activating the EGF receptor (EGFR) signaling pathway [52]. And EGFR was indeed a driver of GalNAc-T activation [49]. Therefore, in cervical cancer, we studied EGFR and its downstream signal transduction pathway, namely PI3K/AKT pathway. PD153035 and LY294002 respectively inhibit EGFR and PI3K, which could inhibit GALNT7′s promoting effect on the proliferation and invasion of cervical cancer cells. Hence, GALNT7 could modulate the EGFR/PI3K/AKT pathway.

Lastly, the results of cervical xenograft models in nude mice revealed that miR-125a-5p inhibited the growth of xenograft tumors, with a significant decrease in tumor size, weight and the protein expression of GALNT7, suggesting that miR-125a-5p suppressed the growth of xenograft tumor cells in vivo.

## Conclusion

In summary, our study showed that miR-125a-5p could inhibit cervical cancer cell growth both in vivo and in vitro. And the potential mechanism might be to inhibit GALNT7 expression and regulate the EGFR/PI3K/AKT pathway. The identification of miR-125a-5p may provide a potential therapeutic target in cervical cancer.

## Data Availability

The datasets used and/or analyzed during the current study are available from the corresponding author on reasonable request. Written informed consents were obtained from each participant involved in this study.
